# Examining the Impact of Trauma-Informed Cognitive Behavioral Therapy on Perinatal Mental Health Outcomes Among Survivors of Intimate Partner Violence (The PATH Study): Protocol for a Feasibility Study

**DOI:** 10.2196/resprot.9820

**Published:** 2018-05-25

**Authors:** Kimberley T Jackson, Sarah Parkinson, Brianna Jackson, Tara Mantler

**Affiliations:** ^1^ Labatt School of Nursing Faculty of Health Sciences The University of Western Ontario London, ON Canada; ^2^ London Health Sciences Center London, ON Canada; ^3^ School of Health Studies Faculty of Health Sciences The University of Western Ontario London, ON Canada

**Keywords:** intimate partner violence, cognitive behavioral therapy, perinatal, mental health, women, nurse

## Abstract

**Background:**

Intimate partner violence (IPV) is a pervasive public health problem, impacting the health and quality of life of survivors worldwide. The trauma of IPV is associated with a high incidence of mental illness, namely depressive and anxiety disorders, and posttraumatic stress disorder (PTSD). Moreover, literature endorses cognitive behavioral therapy (CBT) interventions as a gold standard for those with symptomatology consistent with anxiety disorders, mood disorders, and PTSD. However, efficacy has not been evaluated among a population of pregnant survivors of IPV.

**Objective:**

We present the protocol that will be used to explore the efficacy of trauma-informed cognitive behavioral therapy on maternal and child health outcomes for pregnant women with PTSD, depression, or anxiety symptomatology resulting from IPV. A secondary aim will be to test the validity and feasibility of study methodology to support the successful implementation of a full-scale randomized controlled trial.

**Methods:**

The Promoting Attachment Through Healing (PATH) study will use a mixed-methods approach grounded in an intersectional feminist framework to explore the effectiveness of trauma-informed CBT for pregnant survivors of IPV. Study participants will be recruited through the hospital-based Perinatal Mental Health Clinic (London, Ontario, Canada). A feasibility sample of 20 pregnant women (cohort 1) will be selected to engage in an eight-session antenatal CBT intervention facilitated by the program’s perinatal clinical nurse specialist, with evaluation at baseline, at two months postpartum (intervention and online questionnaire), and at six and twelve months postpartum (online questionnaire only). Concurrently, we will conduct a retrospective audit of 100 medical charts (cohort 2; 50 charts of perinatal women who received CBT and 50 charts of women who did not receive perinatal CBT) from the past five years. The efficacy of the intervention will be based on a reduction of mental illness symptomatology, improved maternal-infant attachment, maternal coping, and maternal quality of life. Additionally, the feasibility of the protocol and acceptability of the intervention from the women’s perspective will be examined. Inductive content analysis of all qualitative data will be used to determine common themes. Additionally, descriptive statistics, including measures of central tendency and dispersion, will be computed for all continuous variables. Alternatively, frequency tables will be constructed for all categorical variables.

**Results:**

The work reported here is in the proposal phase. Once the protocol is implemented, we will report the results in a follow-up paper. Participant recruitment for cohort 1 has started and we have finished data collection for cohort 2. It is anticipated that the results will be available by the end of 2018.

**Conclusions:**

Findings will assess the acceptability of the study methodology and protocol for a full-scale randomized controlled trial. Furthermore, if CBT is proven effective for pregnant survivors of IPV, this intervention could be readily adopted by health care and social support services, thereby contributing to an improved standard of care for this unique population.

**Trial Registration:**

ClinicalTrials.gov NCT03536442; https://www.clinicaltrials.gov/ct2/show/NCT03536442 (Archived by WebCite at http://www.webcitation.org/6zeurv1ay)

**Registered Report Identifier:**

RR1-10.2196/9820

## Introduction

### Background

Intimate partner violence (IPV)—a term that encompasses any act of physical, sexual, or emotional abuse within the context of coercive control by an intimate partner [1]—is a significant public health concern [2]. Intimate partner violence does not discriminate based on socioeconomic status, culture, or religion, and research indicates the global burden disproportionately impacts women [3], with gender-based violence deeply rooted in the patriarchal values espoused throughout history [4]. According to the most recent Statistics Canada profile on family violence, nearly 92,000 cases of IPV were reported to police in 2015, and of those, 79% of survivors were women [5]. While these numbers are deeply unsettling, it is perhaps more alarming that an estimated 1 in 4 Canadian women will be affected by IPV in their lifetime [6].

Ultimately, chronic victimization and coercive control [1] can have truly devastating consequences, including significant mental health challenges that follow survivors across the lifespan [7-9]. A recent meta-analysis and systematic review of 41 studies found women who had experienced IPV were considerably more likely to be diagnosed with a depressive disorder, anxiety disorder, or posttraumatic stress disorder (PTSD) [10]. Importantly, such disorders are more prevalent during pregnancy [10-12], with many studies suggesting the perinatal period poses an elevated risk of IPV [13,14].

Pregnancy is a time of significant physiological change [9]. Newly acquired fatigue, physical pain and discomfort, and emotional irregularity greatly impact one’s ability to cope with environmental stressors and engage in health promotion behaviors [15-17]. When combined with the intimate and invasive nature of perinatal care, it is not surprising that this time period may be traumatic and potentially triggering (in relation to PTSD) for survivors of IPV [18,19]. This is especially salient for those with symptomatology of depression, anxiety or PTSD [7,11,20]. Additionally, the highly-medicalized nature of prenatal interventions, labor and delivery, and postpartum care further contributes to feelings of vulnerability and powerlessness, thereby compromising the mental health and wellbeing of at-risk women [21]. Collectively, these factors substantially increase the likelihood of obstetrical complications including infant prematurity, low birth weight, and developmental delays [22-24]. Furthermore, such trauma and poor mental health are known to negatively impact maternal-child attachment [25], which can significantly affect the development of the child over the course of their lifespan [26-28].

Despite the pervasive nature of IPV and its deleterious health and social sequelae, particularly affecting childbearing women, there remains a considerable dearth of nursing and allied health literature evaluating mental health interventions for this population [29]. There is a critical need for individualized, trauma-informed care to facilitate optimal maternal and child health outcomes across the lifespan [29,30]. Fortunately, viable treatment options, such as cognitive behavioral therapy (CBT) exist to address such challenges; however, research exploring the efficacy of CBT among pregnant populations is lacking. As such, the purpose of the aptly named Promoting Attachment Through Healing (PATH) study is to test the suitability of CBT for the treatment of IPV-related depression, anxiety, and/or PTSD among pregnant women, which can in turn foster meaningful maternal-infant attachment and improve overall health and well-being.

### Objectives

The two overarching goals for this pilot study are: (a) to explore the efficacy of trauma-informed cognitive behavioral therapy (henceforth referred to as CBT) on maternal and child health and attachment outcomes for pregnant women with PTSD, depression, or anxiety symptomatology, resulting from IPV; and (b) to test the feasibility and acceptability of study methodology to support the successful implementation of a full-scale randomized controlled trial. To assess the ability of the proposed research to achieve the desired effect, we present five objectives:

1. To determine the impact of antenatal CBT on PTSD symptomatology among at-risk childbearing women
2. To determine the impact of antenatal CBT on depressive symptomatology among at-risk childbearing women
3. To determine the impact of antenatal CBT on anxiety symptomatology among at-risk childbearing women
4. To determine the impact of antenatal CBT on maternal-infant attachment among at-risk childbearing women
5. To determine the feasibility of the study protocol, by evaluating its process coherence, resource requirements, and perceived value through participant feedback

## Methods

### Design

This pilot project will consist of two cohorts: participants receiving CBT (cohort 1), as well as a retrospective chart audit of individuals who participated in the same CBT treatment within the last five years (cohort 2, intervention arm), and participants who received the accepted standard of care (cohort 2, standard care).

#### Cohort 1

A sample of 20 women currently attending the hospital-based, outpatient Perinatal Mental Health Clinic (London, Ontario, Canada) will be invited to participate in an eight-session antenatal CBT program facilitated by the clinic’s perinatal clinical nurse specialist (PCNS). CBT is currently the gold-standard approach for treating depression and anxiety. What is unique about this intervention is that it is tailored to the antenatal period and integrates attachment theory. Typically, women undergo initial screening at their pre-admission appointment for signs and symptoms of depression, anxiety, and PTSD, as well as for history of IPV. If women meet any of these criteria, they are referred for in-hospital social work or psychiatry consultations as necessary. While this intervention is currently provided to a small subset of women receiving antenatal care at the clinic, its efficacy remains untested. As such, this is considered a “specialty service” provided to identified at-risk women, and is not considered the accepted standard of care at this time.

#### Cohort 2

A retrospective medical chart review of 100 women will be conducted. All women must have received antenatal care at the recruiting hospital within the last five years and screened positive for IPV. This time frame was selected as it is representative of the duration in which the clinic’s current PCNS has been providing CBT to at-risk women. Of the 100 charts selected, 50 women must have participated in the CBT program (intervention arm), while the remaining 50 who did not will be assigned to the standard care group (these participants would have been eligible to participate but were not referred due to either lack of knowledge of the CBT program or a lack of support of the CBT program by the main health care provider). A sample size of 50 for both groups (intervention arm and standard care) will be sufficient to determine the feasibility of the study protocol for a future large-scale randomized control trial (RCT) [31].

### Participant Inclusion Criteria

#### Cohort 1

Inclusion criteria include: 1) English-speaking; 2) pregnant women (any gestational age); 3) living in Ontario; 4) attending the Perinatal Mental Health Clinic at the recruiting hospital; 5) a history of IPV (disclosed to the PCNS during their intake assessment); and 6) symptomatology consistent with PTSD, depression, or anxiety that adheres to the diagnostic criteria outlined in the Diagnostic and Statistical Manual of Mental Disorders (DSM)-V (as assessed by the PCNS at intake).

#### Cohort 2 (Intervention and Standard Care Arms)

The inclusion criteria above apply to the intervention arm of cohort 2 as well. Additional criteria include: 1) received PCNS-delivered CBT at the Perinatal Mental Health Clinic in the past five years; and 2) at least 50% of the data required for the chart data extraction is present.

For the standard care arm, additional criteria include: 1) having received antenatal care (but not PCNS-delivered CBT) at the Perinatal Mental Health Clinic in the past five years; and 2) at least 50% of the data required for the chart data extraction is present.

### Recruitment

#### Cohort 1

Participant recruitment will be facilitated through the PCNS at the London Health Sciences Center (LHSC) in the Perinatal Mental Health Clinic. Women who are receiving antenatal care at the clinic and meet the above eligibility requirements will be provided with a business card, should they wish to learn more about the study. To mitigate any undue influence or pressure to partake in the pilot project, potential study participants will be informed that their involvement is voluntary, and their involvement with the study will have no impact on the nature or quality of care. Participants interested in the study will be asked to contact the research team, at which time the safety protocol will be enacted, and eligibility and recruitment will be completed.

#### Cohort 2 (Intervention and Standard Care Arms)

Recruitment will be facilitated through the health records department at LHSC. The PCNS will identify participants who have received PCNS-delivered CBT in the last five years (intervention arm) and a general chart audit from the same time period will be used for the standard care arm. For both samples, a random sample approach will be undertaken wherein the research assistant (RA) uses a table of random numbers to select charts.

### Safety and Security

Ensuring the physical and emotional safety of women participating in the study is of paramount importance. Upon enrollment in the study, a safety plan will be enacted, outlining secure methods of communication (via email and telephone), information storage, and an emergency protocol if contact with the participant is lost unexpectedly. This particular safety plan has been utilized successfully by both principal investigators in previous research involving survivors of IPV without any adverse events [32]. To further mitigate the risk that perpetrators of violence will discover their partner’s involvement in the study, strict privacy and confidentiality precautions will be adhered to during the transfer and storage of participants’ health information and demographic data. All personal identifiers will be removed and replaced with an encrypted identification code unique to each participant, thus ensuring anonymity. Electronic documents will be stored on the university server behind a secure firewall, while physical copies of interview transcripts and questionnaires will be stored on campus within locked research offices.

Given the sensitive and potentially triggering nature of content raised during structured in-person interviews, all study personnel will be trained to identify acute signs of distress and initiate a stress reaction protocol. This will involve the immediate discontinuation of the interview, followed by referrals to appropriate crisis stabilization and treatment interventions if needed, including the 24-hour helpline for abused women. All participants will be offered a handout describing the signs and symptoms of stress reaction along with various community resources capable of providing comprehensive mental health support.

Finally, should a participant disclose suicidal ideation—whether shared with the PCNS or RA directly, or flagged as high risk (indicated by an affirmative response to questions of self-harm) when completing the Edinburgh Postnatal Depression Scale (EPDS)—they will be immediately referred to the London-Middlesex Suicide Prevention Council Support Line by the individual, or through automatic prompt via the online questionnaire. If it is suspected that juvenile dependents of study participants are experiencing abuse or neglect, members of the research team will report the need for protection to the Children’s Aid Society, as per regulation set forth by the Child and Family Services Act (1990).

### Data Collection

Data collection will occur simultaneously for both cohorts.

#### Cohort 1

Data collection will occur at baseline (T1; prior to the PCNS-delivered CBT and during pregnancy), two (T2), six (T3), and twelve (T4) months postpartum. At T1 and T2, a mixed-method data collection model will be utilized wherein women will complete a semistructured interview for approximately one hour (audio-recorded) and complete a short demographic survey. At T3 and T4, participants will be sent an electronic link to complete a questionnaire (or alternatively, if this is thought to be unsafe, a member of the research team will meet with the participant to complete a hardcopy version).

During the T1 data collection interaction, the study protocol will be thoroughly described and consent to participate will be obtained. Throughout the data collection period (T1-T4), a detailed safety plan will be reviewed (updated as needed), and participants will be provided a $25 honorarium (retail gift card) in recognition of their contribution to the study. To further reduce potential barriers to involvement, those in need of reimbursement for childcare or transportation expenses to attend the in-person interviews will be offered $15 and $10, respectively.

#### Cohort 2

A data abstraction instrument (developed by the co-principal investigators) will be used to collect data. Data will be pulled from multiple sources including: the e-chart, nursing chart, paper chart, and nursing care plan. Maternal data extraction will include: a) scope of mental illness, including diagnosis and current treatments; b) general physical and mental health status; c) obstetrical history; d) birth summary; e) care plan, including CBT interventions; f) evidence of learned CBT strategies or alternative coping strategies used during birth; g) birth experience; h) evidence of maternal-infant attachment; and i) demographics. Conversely, data extraction for infants at the time of parturition will include: a) gestational age; b) weight; c) length; d) head circumference; e) complications and interventions; f) Appearance, Pulse, Grimace, Activity, and Respiration (Apgar) score; and g) newborn physical exam. The chart review will take place at the recruiting hospital and does not require any patient contact. No identifying information will be collected, as the audit will be conducted for each chart in its entirety during a single data extraction period, thereby eliminating the need to refer back to any particular patient’s chart.

### Outcome Measures

All interviews and questionnaires seek to understand the lived experience of IPV within the context of pregnancy and childbirth, as well as the impact of CBT on the five primary study objectives described previously. Specifically, it is expected that this study will ascertain the impact of CBT on a) maternal mental health*,* and b) maternal-infant attachment. These outcomes will be measured using the PTSD Checklist (PCL-5), EPDS, Spielberger State-Trait Anxiety Inventory (STAI), World Health Organization Quality of Life Brief Assessment (WHOQOL-BREF), Maternal Attachment Inventory (MAI) and Proactive Coping Inventory (PCI). Each will be discussed in turn.

#### PTSD Checklist (PCL-5)

The PCL-5 is a PTSD Checklist for DSM-V symptomatology consisting of 20 items in a standardized self-report measure that assesses the presence and severity of PTSD [33]. Respondents indicate how much they have been bothered by a particular symptom over the past month using a 5-point Likert scale, with responses varying between 0 (not at all) and 4 (extremely). A total symptom severity score—ranging from 0 to 80—is obtained by summing the self-reported ratings for all 20 items, with higher scores indicating greater PTSD symptomatology. Based upon thorough psychometric evaluation [33], the PCL-5 has demonstrated excellent internal consistency (alpha=.94) and construct validity.

#### Edinburgh Postnatal Depression Scale

Postpartum depression will be measured using the EPDS—a 10-item screening questionnaire used to identify symptoms of a depressive disorder, as outlined in the DSM-5 [34]. Women are asked to answer each question by selecting the response that best describes their feelings during the previous 7 days. Responses are assigned points from 0 to 4, with higher values corresponding to more severe depressive symptomatology. Points from each question are summed, with total scores ranging from 0 to 30. The EPDS has been used consistently among perinatal practitioners since its inception, and is widely recognized as a valid and reliable screening tool with strong internal consistency (alpha=.83-.87) [35]. The EPDS has a cut score of 15 or more for antenatal women indicating a major depressive episode.

#### Spielberger State-Trait Anxiety Inventory

Anxiety among at-risk women will be assessed using the STAI [36]. The STAI is an introspective psychological instrument consisting of 40 self-reported items, separated into two subscales (ie, A-State and A-Trait), each with 20 items. “State anxiety” is defined as a transient momentary emotional status that results from situational stress, while “trait anxiety” represents a predisposition to react with anxiety in stressful situations [37]. All items are scored using a 4-point Likert scale (1-4), with totals ranging from 20 to 80 for each subscale, with higher scores suggestive of greater anxiety. Considerable evidence [36] attests to the construct and concurrent validity of the STAI (alpha=.86-.91).

#### World Health Organization Quality of Life Brief Assessment

Maternal quality of life (QOL) will be assessed using the WHOQOL-BREF, an instrument comprised of 26 items measuring the following four domains: physical health, psychological health, social relationships, and environment. Responses for each question range from 1 to 5 along a Likert-type scale, with higher scores corresponding to a greater self-perceived QOL. The WHOQOL-BREF has been found to have strong discriminant validity (*r*>.45) and internal consistency (alpha=.84) [38].

#### Maternal Attachment Inventory

Maternal-infant attachment will be evaluated using the MAI [39]. The MAI is a 26-item self-report instrument is measured on a four-point Likert scale, ranging from a) almost always, b) often, c) sometimes, and d) almost never. A total score is obtained by converting subjects’ responses to corresponding numeric values as follows: a) 4 points, b) 3 points, c) 2 points, and d) 1 point. All items are summed, resulting in scores ranging from 26 to 104. Higher scores indicate stronger feelings of attachment toward the newborn child. This instrument was initially tested in a sample of 196 women at one month postpartum, resulting in acceptable internal consistency and validity (alpha=.85) [39].

#### Proactive Coping Inventory

Maternal coping will be measured using the PCI [40], a multi-dimensional coping scale that assesses how well individuals utilize resources in the development of positive coping strategies to combat distress. The PCI is comprised of seven subscales, each of which assesses a unique dimension: proactive coping, preventive coping, reflective coping, strategic planning, instrumental support seeking, emotional support seeking, and avoidance coping. All items are assessed on a 4-point Likert scale, ranging from 1 (not at all true) to 4 (completely true). Responses are summed to obtain a total score for each subscale, with higher scores reflective of greater proactive coping skills. All subscales were found to have good validity and high internal consistency (alpha=.71-.85) [40].

#### Feasibility

Feasibility of the intervention and protocol will be evaluated through the semistructured interviews with women in cohort 1. To ascertain feasibility, open-ended questions will be asked to evaluate fidelity and resource requirements. Fidelity will be assessed by examining the five core components of the intervention with global open-ended questions for each, such as:

1. What have you learned about the brain (and the brain during pregnancy)?
2. What have you learned about the nature of trauma and its effect on the brain?
3. What have you learned about the effects trauma can have on your attachment with your baby?
4. What have you learned about triggers?
5. What tools have you learned about to manage triggers?

Resource requirements will be assessed from: 1) a provider perspective by tracking the length of the CBT sessions and the number of sessions; 2) a patient perspective by tracking any costs incurred to participate in the CBT session (eg, travel, childcare, etc); and 3) a system perspective by tracking the use of facilities and referrals to other resources.

#### Acceptability

To assess acceptability of the intervention, we will ask four global open-ended questions assessing the perceived value of the intervention including:

1. What strategies do you see yourself using during labor/delivery and beyond?
2. How has your CBT work impacted how you feel about labor/delivery and beyond?
3. How has your CBT work impacted the way you feel towards your unborn child? Yourself? As a woman?
4. Would you recommend this intervention to someone else in a similar situation as you? Why or why not?

### Statistical Analysis

For quantitative data, the overall impact of the PCNS-facilitated CBT intervention will be assessed using generalized estimating equations [41] to determine the effectiveness over time, and in contrast to standard care. Descriptive statistics, including measures of central tendency and dispersion, will be computed for all continuous variables and frequency tables will be constructed for all categorical variables.

#### Qualitative Analysis

For qualitative data using NVivo Software, an inductive content analysis will be executed to determine common themes [42]. This methodology supports a holistic and rich description of data, ideally suited to address project goals. This analysis will be grounded in an intersectional feminist framework [43]. This framework emphasizes that gender-based violence needs to be understood within the context of how identity and social location intersect and interact and how systems of power, oppression, and privilege shape women’s lives [44,45]. Analysis will take place at the end of data collection and will be conducted independently and simultaneously by 2 investigators and a research assistant. NVivo software will support analysis through coding and providing a comprehensive data management system. Each coder will become immersed in the data and once saturation is reached coding will commence [46]. Initially, open coding data line by line will be used and when themes emerge they will be organized into similar topics and then categories. Each category will be examined to determine meaning and will be assigned a working name and flexible definition. Axial coding will then be completed by recontextualizing the data that was coded into each category to determine consistency within the category (to decrease human bias in selective coding). Lastly, the categories will be re-examined to finalize the name and definition as well as to ascertain the underlying meaning of the theme within the context of the findings. Once each coder is finished coding, findings will be compared to ascertain if there is agreement on major emergent themes. If there is no agreement, a majority will be used to classify major themes.

### Power

#### Cohort 1

A sample of 20 women was selected as this is a feasibility study and it is anticipated we will reach saturation (assessed by the sole interviewer with the use of field notes) in the qualitative data [46]. This sample will be used to inform future power analysis for the large-scale RCT.

#### Cohort 2

One hundred charts will be audited (50 from each of the two arms, the intervention and standard care arms). This sample size, for this feasibility study, was selected as it will allow us to extend the breadth and depth of analysis from cohort 1. This ultimately enhances the validity of the study’s findings [31] by contextualizing the differences between the intervention and standard care, therefore contributing to valuable knowledge translation.

## Results

The work reported here is in the proposal phase. Once the protocol is implemented, we will report the results in a follow-up paper. However, we hypothesis that PCNS-delivered CBT will result in decreased PTSD, anxiety, and/or depression symptomatology, and improved maternal-infant attachment, coping, and QOL. We also hypothesize this study will be feasible in terms of fidelity and deliverability, as well as be highly acceptable to participants.

## Discussion

### Study Rationale and Future Direction

We hypothesize that PCNS-delivered CBT will be effective in treating PTSD symptomatology, depression and anxiety disorders affecting the sample of pregnant women selected [47], thereby contributing to participants’ improved QOL and maternal-infant attachment, and positive child health outcomes following birth.

The PATH study serves to build upon existing health literature that explores the positive impact of CBT for survivors of IPV and those experiencing mental health challenges [48-50] within periods of transformational change associated with pregnancy, childbirth, and motherhood. This feasibility study will help to address a current lack of literature in the area by filling a gap in intervention research aimed at improving the health and QOL of perinatal women who have experienced IPV and have symptomology consistent with PTSD, depression and/or anxiety.

Furthermore, successful implementation will help to determine the feasibility and acceptability of the study methodology and protocol, to support the future full-scale RCT. Efficacy will be determined in relation to a reduction in PTSD, anxiety, and depressive symptoms, and improvement in maternal-infant attachment, maternal coping, and maternal QOL. Feasibility will be assessed in relation to fidelity and resource allocation (from the provider, participant, and system perspectives) and acceptability will be measured in terms of perceived value from the participants’ perspective.

### Limitations

The proposed study is not without limitations, namely the small sample size, limited recruiting methodology, and high reliance on the PCNS who is delivering CBT at the recruitment site. The small sample size chosen for this feasibility study is a limiting factor for statistical analyses as well. The fact that recruiting is limited to a single site could also be problematic as this significantly decreases the pool of potential participants. Finally, the high reliance on the PCNS who is delivering CBT at the recruitment site also limits the pool of participants, in addition to potential concerns regarding scale-up and reproducibility of the intervention. While having one intervention decreases the variability in terms of consistent delivery of the intervention, it speaks to a potential difficulty in ascertaining if the results were due to the intervention alone or the intervention/interventionist.

### Conclusions

This is the first intervention research of its kind examining the impact of perinatal CBT for women who have experienced IPV with mental illness. If the hypotheses of this research are correct, then not only would that support the scale-up of the intervention, it could also have far-reaching implications for individuals and society. Specifically, the findings would not only benefit individual participants and their families in terms of strengthened maternal-infant bonds and family units, but may also result in broader societal benefits, including reduced chronic health care utilization and associated treatment costs, as well as increased productivity, participation, and economic contribution within the London community [51,52].

## Appendix

Multimedia Appendix 1 Peer-reviewer report.

## Abbreviations

Apgar score: Appearance, Pulse, Grimace, Activity, and Respiration score
CBT: cognitive behavioral therapy
DSM-V: Diagnostic and Statistical Manual of Mental Disorders—Fifth Edition
EPDS: Edinburgh Postnatal Depression Scale
IPV: intimate partner violence
LHSC: London Health Sciences Centre
MAI: Maternal Attachment Inventory
PATH: Promoting Attachment Through Healing
PCI: Proactive Coping Inventory
PCL-5: PTSD Checklist for DSM-5
PCNS: perinatal clinical nurse specialist
PTSD: posttraumatic stress disorder
QOL: quality of life
RA: research assistant
RCT: randomized control trial
STAI: State-Trait Anxiety Inventory
WHOQOL-BREF: World Health Organization Quality of Life Brief Assessment
