# Supplementary material for: Examining the Impact of Trauma-Informed Cognitive Behavioral Therapy on Perinatal Mental Health Outcomes Among Survivors of Intimate Partner Violence (The PATH Study): Protocol for a Feasibility Study
Source: JMIR Res Protoc. 2018 May 25;7(5):e134. doi: 10.2196/resprot.9820 (PMC5993975; doi:10.2196/resprot.9820)
Supplement: Multimedia Appendix 1 [file resprot_v7i5e134_app1.pdf]

WOMEN'S COLLEGE HOSPITAL
women's xchange

$15K Challenge
Decision
Committee

Summary of Committee Report

**Name of Principal Co-Applicant: Kimberley Jackson and Tara Mantler**
**Project Title: Examining the impact of trauma-informed cognitive behavioural therapy among at-risk pregnant women on perinatal mental health outcomes: A pilot study**
**RFP Deadline: Oct 3, 2016**

Review Committee Comments:

This proposal is well-articulated and has significant scientific merit. The methods align well with the study objectives and the knowledge translation plan is strong. Some elements of this proposal are unclear and require further details, including the analysis plan. The committee also notes there is a lack of community involvement in this study, and advises inclusion of a community partner in shaping the research.

Decision:

Funding Approved.

If you choose to accept this funding opportunity from Women's Xchange, please read, sign and scan the attached Award Letter.

Please return letter to:

info@womensxchange.ca

If you have any questions or comments, please contact Sagal Abdulle at:

(416) 351-3732 ext 3755
Sagal.abdulle@wchospital.ca
